# Efficacy and safety of acupuncture therapy for asymptomatic infection of COVID-19: A protocol for systematic review and meta-analysis: Retraction

**DOI:** 10.1097/MD.0000000000025227

**Published:** 2021-03-19

The article “Efficacy and safety of acupuncture therapy for asymptomatic infection of COVID-19: A protocol for systematic review and meta-analysis”,^[1]^ which appeared in Volume 99, Issue 41 of Medicine, is being retracted due to self-duplication. An investigation into the paper and a second protocol published in Medicine^[2]^ with shared authors found the language and aim of both papers were similar enough to constitute duplication.
